# Review of Prognostic Expression Markers for Clear Cell Renal Cell Carcinoma

**DOI:** 10.3389/fonc.2021.643065

**Published:** 2021-04-28

**Authors:** Florent Petitprez, Mira Ayadi, Aurélien de Reyniès, Wolf H. Fridman, Catherine Sautès-Fridman, Sylvie Job

**Affiliations:** ^1^Programme Cartes d'Identité des Tumeurs, Ligue Nationale Contre le Cancer, Paris, France; ^2^Centre de Recherche des Cordeliers, INSERM, Sorbonne Université, Université de Paris, Equipe Inflammation, Complément et Cancer, Paris, France

**Keywords:** prognostic markers, clear cell renal cell carcinoma (ccRCC), multivariate analysis, independent datasets, cox models

## Abstract

**Context:** The number of prognostic markers for clear cell renal cell carcinoma (ccRCC) has been increasing regularly over the last 15 years, without being integrated and compared.

**Objective:** Our goal was to perform a review of prognostic markers for ccRCC to lay the ground for their use in the clinics.

**Evidence Acquisition:** PubMed database was searched to identify RNA and protein markers whose expression level was reported as associated with survival of ccRCC patients. Relevant studies were selected through cross-reading by two readers.

**Evidence Synthesis:** We selected 249 studies reporting an association with prognostic of either single markers or multiple-marker models. Altogether, these studies were based on a total of 341 distinct markers and 13 multiple-marker models. Twenty percent of these markers were involved in four biological pathways altered in ccRCC: cell cycle, angiogenesis, hypoxia, and immune response. The main genes (*VHL, PBRM1, BAP1*, and *SETD2*) involved in ccRCC carcinogenesis are not the most relevant for assessing survival.

**Conclusion:** Among single markers, the most validated markers were *KI67, BIRC5, TP53, CXCR4*, and *CA9*. Of the multiple-marker models, the most famous model, ClearCode34, has been highly validated on several independent datasets, but its clinical utility has not yet been investigated.

**Patient Summary:** Over the years, the prognosis studies have evolved from single markers to multiple-marker models. Our review highlights the highly validated prognostic markers and multiple-marker models and discusses their clinical utility for better therapeutic care.

## Introduction

Clear cell renal cell carcinoma (ccRCC) is the most common histological subtype of kidney cancers, accounting for around 85% of all renal cell carcinomas (1). Although localized ccRCC can be treated by partial or total surgical ablation of the kidney, advanced ccRCC remains a clinical challenge, with 5-year overall survival (OS) rates of 0–20% (2). Over 90% of ccRCC cases have undergone a loss of heterozygosity of the chromosome 3p, where notably *VHL* is located. Moreover, *VHL* is mutated in 70% of ccRCC tumors and hypermethylated in 15% (3), and inactivating *VHL* mutation is considered the main driver of ccRCC carcinogenesis (4). Loss of *VHL* leads to activation of hypoxia-inducible factors and subsequently to vascular endothelial growth factor (VEGF)-mediated angiogenesis (5). Therefore, tyrosine kinase inhibitors with antiangiogenic properties have become a crucial treatment option for ccRCC patients (6).

Over the past decades, a large series of studies have aimed at finding prognostic markers for ccRCC in order to identify patients who were at higher risk of relapse and death. During this time, technologies have largely evolved—from surface proteins measured by single-molecule immunohistochemistry (IHC) to reverse-transcription quantitative polymerase chain reaction (RTQ-PCR) for mRNA and RNA sequencing (RNA-seq) for mRNA or long non-coding RNAs. Studies gradually incorporated more cases, while assessing an ever-larger number of putative targets, many focusing on angiogenesis-related targets, especially in the context of antiangiogenic therapies (7). Others emphasized immune-based approaches (8), as immune infiltration of tumors is a common prognostic factor in many different types of malignancies (9). Here, we aimed at reviewing prognostic markers that have been proposed for ccRCC during the 15 years between 2003 and 2018 through a thorough analysis of over 2,700 records from the literature.

## Materials and Methods

### PubMed Query

A literature search was carried out using PubMed database to identify prognostic expression markers from studies published between 2003 and 2018. The PubMed query was: (clear cell renal cell carcinoma) AND (prognosis OR cancer prognosis OR cancer survival) AND (human OR *Homo sapiens*) AND (expression OR transcription OR transcriptome OR immunohistochemistry OR IHC). The search was conducted in December 2018.

### Study Selection

**Figure 1:**
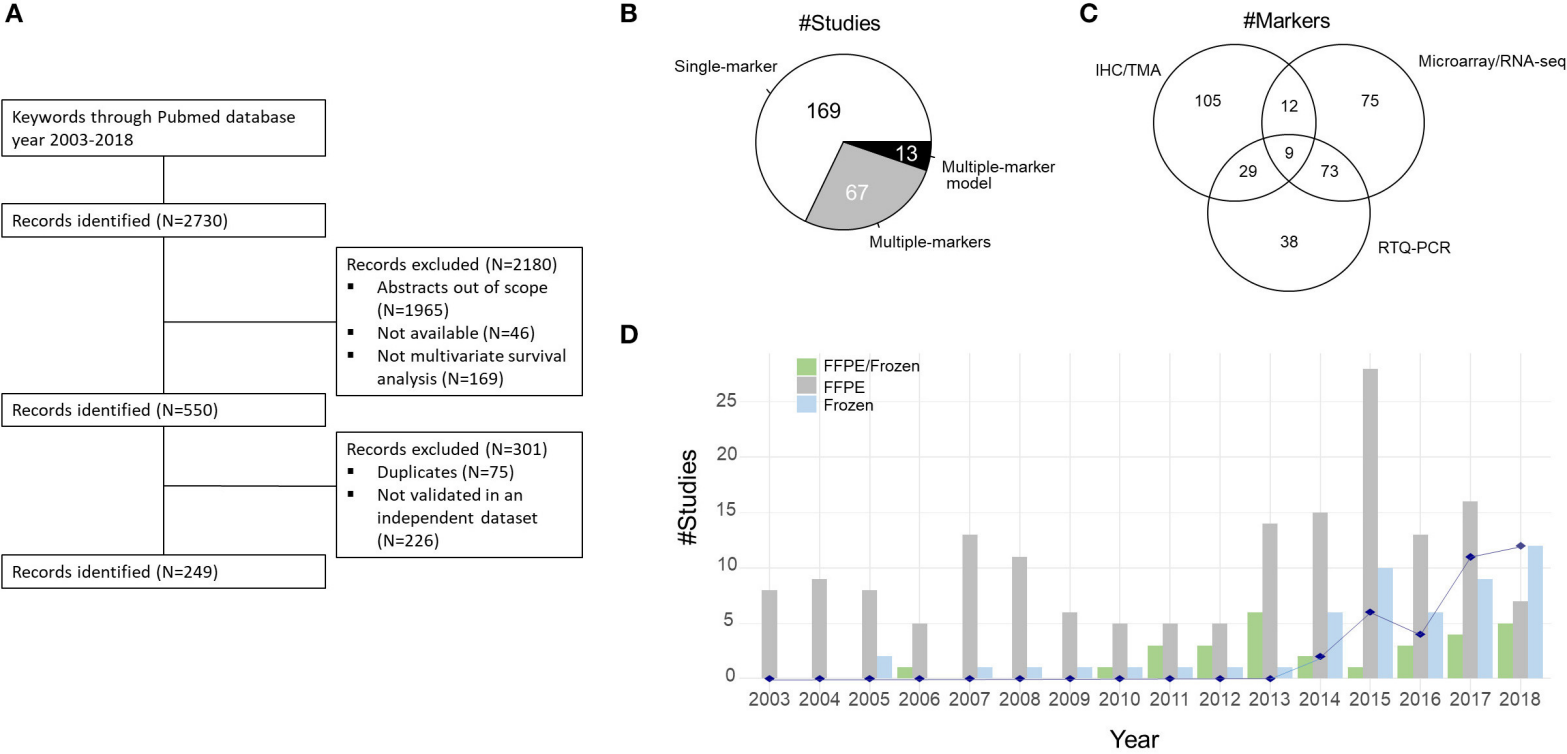
**(A)** Consort diagram showing the selection process of studies included in the literature review. **(B)** Distribution of the studies investigating one marker, several markers, or multiple-marker models. **(C)** Venn diagram of the distribution of technologies used to quantify the expression level of the 341 genes. IHC, immunohistochemistry; TMA, tissue microarray; RNA-seq, RNA sequencing; RTQ-PCR, reverse-transcription quantitative polymerase chain reaction. **(D)** Distribution of the number of studies according to the type of biomaterial over the years: Frozen samples and/or formalin-fixed paraffin-embedded (FFPE) samples. The blue line indicates the number of studies using The Cancer Genome Atlas (TCGA) dataset as training or validation dataset.

The following inclusion criteria were applied: original article (not reviews, editorials, conference abstracts); English language; research was performed on human ccRCC tissue samples; and association of the expression level of candidate genes with patient survival was investigated in multivariate analyses in several independent datasets. The following patient survivals were considered: OS, progression-free survival (PFS), relapse-free survival (RFS), disease-free survival (DFS), cancer-specific survival (CSS), and metastasis-free survival (MFS). The studies describing transcriptome-based clusters of samples associated with patient survival were included. Case reports were excluded as well as studies performed on metastatic or advanced cohorts only. Two authors (SJ and FP) evaluated the titles and the abstracts of all 2,730 publications identified by the search strategy, and all 550 publications thought to be potentially relevant were retrieved in full (Figure 1A). The same authors then assessed full publications for eligibility. Any study was included in the review with the agreement of both authors.

### Statistical Analysis

The analyses were performed using R software version 3.5.2. We carried out hypergeometric tests on signaling pathways of three databases [Kyoto Encyclopedia of Genes and Genomes (KEGG), Gene Ontology (GO), and Reactome] using the reviewed prognostic markers. We used the gene lists with the most significant hypergeometric test *p*-values to illustrate the prognostic markers within the four mentioned pathways: angiogenesis (GO), hypoxia (Reactome), cell cycle (KEGG), and immunity (Reactome).

## Results

### Literature Evaluation

The PubMed query identified 2,730 publications (Figure 1A). Upon review, 2,180 publications were first excluded after title and abstract reading as being irrelevant to the present study, not available, or lacking validation. Of the remaining 550 publications, 301 studies were excluded due to the absence of validation on independent datasets or because of duplicate publication. Thus, the final total number of studies included in the present review was 249 (Figure 1A and Supplementary Table 1).

### Collection of Prognostic Markers

The 249 selected studies reported 341 distinct prognostic markers, 321 related to coding genes (mRNAs/proteins) and 20 to non-coding RNAs (six long non-coding RNAs, 14 microRNAs). While 169 out of the 249 studies (67.9%) focused on the prognostic impact of a single marker, the 80 remaining studies integrated multiple-marker analyses, 13 of them providing mathematical models computing a risk score (Figure 1B). Forty-one percent of markers (45/111) used in single-marker analyses were integrated in multiple-marker models. In the original publications, the expression levels of the prognostic markers were characterized using different technologies mainly represented by IHC, tissue microarray (TMA), RTQ-PCR, microarrays, and RNA-seq technologies (Figure 1C). These technologies exploit different types of biomaterials: IHC/TMA technologies generally use formalin-fixed paraffin-embedded (FFPE) samples and quantify a marker at a proteomic level, while RTQ-PCR, microarrays, and RNA-seq use frozen samples and quantify markers at a transcriptomic level. IHC and TMA were the most common identification methodologies used (143/341 targets, 41.9%). Among the 123 out of the 341 markers (36%) identified by two or more methodologies, 50 markers were validated both at the protein level (by IHC/TMA) and at the RNA level (by RTQ-PCR, RNA-seq, or microarrays) (Supplementary Table 1).

Over the years, we have remarked an increase in the number of analyzed frozen samples (Figure 1D). This increase is linked, on the one hand, to the growing accessibility to high-throughput technologies (microarrays and then RNA-seq) and, on the other hand, to the public datasets available in genomics data repositories such as Gene Expression Omnibus (GEO), array express, or the GDC data portal of The Cancer Genome Atlas (TCGA) Program. Eighty percent of studies using public datasets as training and/or validation sets use TCGA cohort composed of 532 ccRCC samples (Figure 1D). Our review excluded studies resulting from the analysis of familial cohorts and of advanced or metastatic cohorts. The studied cohorts essentially included unselected samples from ccRCC patients who have had a radical or partial nephrectomy, and the samples are primary tumors.

### Main Biological Pathways Related to Prognostic Markers of Clear Cell Renal Cell Carcinoma

**Figure 2:**
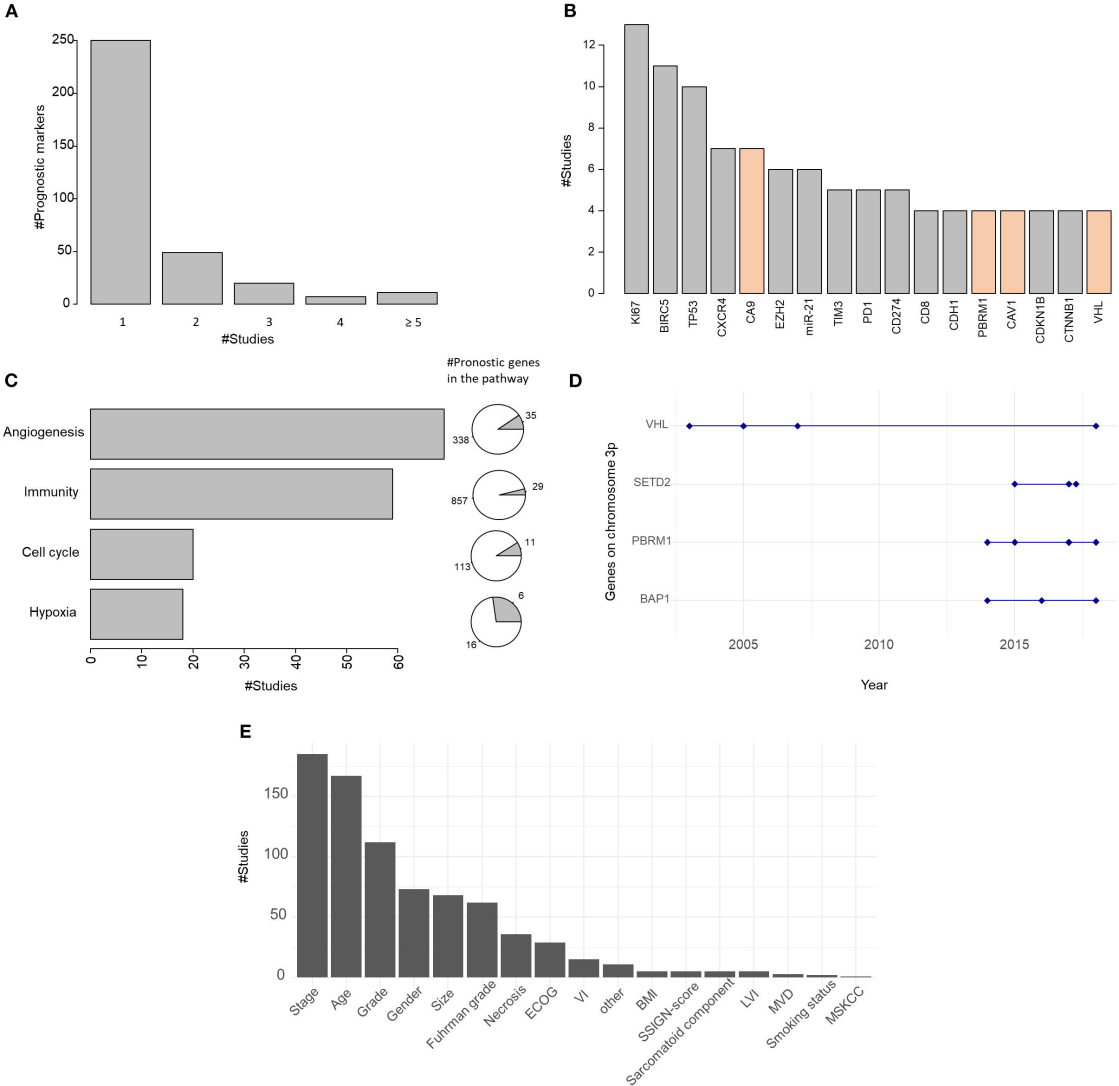
**(A)** Barplot of the number of markers cited in one or more studies. **(B)** Barplot of the most investigated prognostic markers. In orange are indicated prognostic markers specific to clear cell renal cell carcinoma (ccRCC). **(C)** Barplot of the number of studies investigating markers involved in the main biological pathways: angiogenesis, immunity, cell cycle, and hypoxia. Pies on the right represent the proportion of prognostic markers in the pathway. **(D)** Distribution of the studies assessing the prognostic value of genes on chromosome 3p over the years. **(E)** Barplot of the number of studies integrating clinical covariates. ECOG, Eastern Cooperative Oncology Group; VI, vascular invasion; BMI, body mass index; SSIGN, Stage, Size, Grade, and Necrosis; LVI, lymphovascular invasion; MVD, microvessel density; MSKCC, Memorial Sloan Kettering Cancer Center.

As previously stated, we excluded markers identified in a single study on a single cohort. Among the 341 prognostic markers, 250 markers were validated on internal datasets (Figure 2A). The 86 markers confirmed in two or more independent studies can be found in Supplementary Table 2. Seven markers were found in six or more studies (Figure 2B): KI67 (10–22), *BIRC5* (23–32), *TP53* (14, 18, 21, 33–39), *CXCR4* (40–46), *CA9* (47–53), *miR-21* (54–59), and *EZH2* (60–65). Some of them exceed the mere field of ccRCC. For instance, *BIRC5* is able to inhibit cell death and is upregulated in most, if not all, cancers (66); *TP53* is implicated in DNA damage repair and is mutated in a large portion of cancers (67). Of the 17 most reported prognostic markers, some are more tightly related to ccRCC, such as *VHL* (68–71), *PBRM1* (72–75)*, CA9* (47–53), or *CAV1* (76–79).

Of the 341 reviewed prognostic markers, 20% are involved in biological pathways altered in ccRCC (80) of which the main ones are cell cycle, angiogenesis, hypoxia, and immune response (Figure 2C), which are involved in classical hallmarks of cancer (81).

#### Hypoxia and Angiogenesis

*VHL* inactivation, through mutation, hypermethylation, and/or loss of heterozygosity (3), is regarded as the key genetic event leading to ccRCC formation (4). This dysregulation of the Von Hippel–Lindau pathway leads to HIF1α-HIF1β transcription factor activation and increased hypoxia response and neoangiogenesis through VEGF signaling. Expectedly, hypoxia and angiogenesis pathways are importantly represented in the list of prognostic markers that have been identified in the literature (Figure 2C). Sixty-nine studies listed at least one gene associated with angiogenesis, and 35 such genes were identified: *AAMP* (82), *ANPEP* (83), *APOLD1* (82), *B4GALT2* (84), *C5* (85), *CAV1* (76–79), *CCL2* (86, 87), *CCR2* (87), *CEACAM1* (82), *CTGF* (83), *CTNNB1* (88–91), *CX3CL1* (82), *CXCL10* (92), *CXCL12* (42), *CXCR2* (93), *CXCR4* (40–46), *EPAS1* (94–96), *FLT1* (14), *GPX1* (82), *HIF1A* (52, 97, 98), *HPSE* (99, 100), *IL6* (82), *JAG1* (101, 102), *MMP2* (103), *NOS3* (82), *NOTCH1* (102, 104, 105), *NRP1* (95), *PDGFRB* (106, 107), *PTEN* (108–110), *SERPINE1* (111–113), *SETD2* (74, 114, 115), *TGFBR2* (116, 117), *THSD7A* (118), *VASH1* (100, 119), *VEGFA* (50, 120, 121). Six markers of hypoxia [*ARNT* (95), *CA9* (47–53), *EPAS1* (94–96), *HIF1A* (52, 97, 98), *VEGFA* (50, 120, 121), and *VHL* (68–71)], representing about 30% of the genes involved in the hypoxia pathway, are reported as prognostic markers in 18 publications. Of note, three of these markers (*EPAS1, HIF1A*, and *VEGFA*) also belong to the angiogenesis pathway.

#### Immunity

ccRCC tumors exhibit a rather low mutational burden compared to other tumor types (122). Nonetheless, they have been one of the first tumor types for which immunotherapy with high-dose IL-2 has proved efficient (123), although their responsiveness to immune checkpoint blockade remains rather low, below 30% (124). Moreover, immunity has been repeatedly associated with clinical outcome for this pathology (8). We found 59 articles identifying at least one gene related to immunity (among other pathways) as a prognostic factor in ccRCC. Twenty-nine such genes were identified: *AKT1* (77, 108), *ANAPC5* (125), *ARF1* (82), *BCL2* (39, 126), *C5* (85), *CARD9* (127), *CCR2* (87), *CD274* (26, 128, 128–130), *CD4* (118, 131, 132), *CD44* (100, 133), *CDH1* (63, 103, 134, 135), *CDK1* (136), *CDKN1A* (17, 18, 137), *CDKN1B* (138–141), *CIITA* (127), *DEFB1* (83, 142), *ICOS* (118, 143), *IKBKE* (144), *IL5RA* (145), *IL6* (82)*, MDM2* (34), *NCF2* (127), *PAK1* (146), *PSMD9* (18, 147), *PTEN* (108–110), *RCHY1* (148), *TLR3* (95), *VCAM1* (95, 149), and *VHL* (68–71) (Figure 2C). Some of them were independently reported by several publications. For instance, *CXCR4* (40–46), which encodes a receptor for the lymphocyte chemoattractant CXCL12, was identified by seven publications. *ICOS*, a T cell co-stimulatory molecule, was reported as prognostic biomarker by two publications.

#### Cell Cycle

Expression of cell cycle-related gene signatures or proteins is generally a marker of the presence of highly proliferative cells and is therefore widely regarded as a biomarker of aggressive malignancy and poor prognosis (150, 151). Here, we have observed 11 such genes, reported in 20 publications: *ANAPC5* (125), *CCND1* (18), *CDC7* (125), *CDK1* (136), *CDKN1A* (17, 18, 137), *CDKN1B* (138–141), *CDKN1C* (18), *CDKN2A* (18, 152), *GADD45G* (127), *MDM2* (34), and *TP53* (14, 18, 21, 33–39).

#### Focus on Clear Cell Renal Cell Carcinoma Genes on Chromosome 3p

Over 90% of sporadic ccRCC displays a deletion of chromosome 3p. The ccRCC key event is the alteration of the tumor-suppressor gene VHL (3p25-p26). Its prognostic impact was mainly studied a few years ago (between 2003 and 2007; Figure 2D), but the results were not very significant and often associated with specific subcellular locations (69, 70). Its validation as a prognostic marker was then neglected until recently in a study investigating the cumulative roles of *PBRM1* and *VHL* as risk factors (71).

Whole exome sequencing helped to identify the three other frequently mutated genes on chromosome 3p: *PBRM1* (~40%), *SETD2* (~12%), and *BAP1* (~10%) (71). Several studies investigated their prognostic values since 2014. Studies about *PBRM1* (71, 72, 74, 75) were contradictory. While authors of the study (75) validate PBRM1 as an independent predictor of PFS but not of OS, Jiang et al. (74) and Högner et al. (71) showed opposite results. The prognostic value of *BAP1* was dependent on the cellular localization (153) but was only validated by bivariate Cox models (154) or in combination with the expression of *PBRM1* (72). Finally, *SETD2* was studied in three studies (74, 114, 115) in combination or not with the expression of H3K36me3, and all results agreed with the prognostic role of *SETD2*.

In conclusion, despite the high rate of alterations of these four genes on chromosome 3p and their role in ccRCC carcinogenesis and progression, their prognostic value may be ambiguous, explaining why these markers were not in the most reported prognostic markers, except VHL.

### Prognostic Molecular Markers and Clinical Covariates

Our review focused on independent prognostic markers, meaning that their prognostic impacts evaluated by Cox models remain significant after inclusion of other clinical and/or molecular covariates. Here, 242 out of the 249 studies integrated one or more clinical covariates, while seven studies (22, 74, 121, 130, 132, 155, 156) concluded the independence of the prognostic markers only from comparisons with other molecular markers. The studies using one or more bivariate Cox models were integrated in our review despite the lack of a global multivariate Cox model. The selected studies focused on different types of survival: OS, CSS, PFS, DFS, RFS, or MFS. When filled in, the starting times used to compute survival data may also differ between studies using either date at diagnosis or date at surgery. All these aspects make a direct comparison of prognostic values difficult.

Figure 2E summarizes the clinical covariates used in the 242 studies. The most represented clinical covariates are age, stage, and grade in adequacy with their known prognostic value. The clinical covariates specific to ccRCC such as Fuhrman grade or Stage, Size, Grade, and Necrosis score (SSIGN score) were also represented but to a lesser extent.

The authors of the reviewed studies present multivariate Cox models in the goal to validate the independence of the molecular predictors they studied. In our point of view, they validate above all the use of clinical and molecular covariates to better predict the survival of patients with ccRCC.

### Risk Multiple-Marker Models

Eighty out of the 249 selected studies investigated the prognostic value of several markers. We distinguish multi-marker analyses (*n* = 67 studies; Figure 1B) evaluating several independent predictors in multivariate Cox models and multiple-marker model analyses (*n* = 13 studies) providing a mathematical model that computes a risk score (36, 54, 55, 57, 82, 83, 95, 125, 135, 136, 149, 157, 158). These multiple-marker models were calibrated against a given technology used to quantify expression values (RTQ-PCR, RNA-seq, or nanostring) and used from 2 to 34 markers (Supplementary Table 3). Ten out of these multiple-marker models provide a mathematical formula, represented by a weighted sum of the expression values of each prognostic marker with or without clinical covariates. Three studies provided models based on microRNA expression (54, 55, 57). Mlcochova et al. (135) focused on genes involved in epithelial–mesenchymal transition, and Yang et al. (136) computed a risk model using genes in interaction with the nucleotide degrading enzyme gene *RNASEH2A*. Two unsupervised classifications based on whole transcriptome proposed prognostic ccRCC subtyping (157, 158). Brannon et al. (157) identified two subtypes (ccA and ccB), and Beuselinck et al. (158) proposed four subtype names ccrcc1 to ccrcc4. The two classifications identified subtypes related to a worse prognosis (Brannon: ccB; Beuselinck: ccrcc1 and ccrcc4). The classification by Beuselinck et al. (158) was also related to response to antiangiogenic treatment by sunitinib. The now well-established clinico-molecular prognostic model (95), ClearCode34, was built from the classification by Brannon et al. (157) added to clinical covariates (stage and Fuhrman grade). It is the single risk model using clinical and molecular markers.

### Sub-selection of Prognostic Markers for Clinical Utility

We propose a sub-selection of the most validated prognostic predictors with a potential clinical utility. About the single prognostic markers, we focused on the prognostic markers validated on more than seven independent studies (Figure 2B), validated on fewer technologies based on FFPE and frozen samples. We imposed a prognostic impact independent of the classical clinical covariates (stage, Fuhrman grade, age, gender, and grade) on a large set of samples. The five markers (KI67, BIRC5, TP53, CXCR4, and CA9) validated on seven or more independent studies were validated on FFPE and frozen samples and compared with a large set of clinical covariates. KI67 and BIRC5 remained the more confident given their validation on more than 5,000 samples. The quantification of KI67 was used as secondary objective in two clinical trials on ccRCC patients (NCT03575611 and NCT01253668) to evaluate response to treatment. Three clinical trials in ccRCC patients (NCT02787915, NCT00197860, and NCT01924156) integrated the use of BIRC5 to assess survival or response to BIRC5-loaded dendritic cell vaccines.

We have been less strict on the sub-selection of multiple-marker models, as these models are newer. We focused on gene models validated at least in one independent study (Supplementary Table 3) and whose clinical use has been evaluated in a clinical trial. Six out of the 13 multiple-marker models were validated in external datasets, but only the clinical utility of the 16-gene assay of Rini et al. (82) and the ccrcc classification of Beuselinck et al. (158) were tested in a phase III (NCT00375674) and a phase II (NCT02960906) clinical trial, respectively.

### Discussion

In this review, we conducted an extensive analysis of the literature on prognostic markers in ccRCC over the last 15 years. Published studies evolved according to technological progresses. The oldest studies mainly focused on the prognosis impact of single genes known to be involved in the ccRCC carcinogenesis such as VHL or HIF1A, mostly validated by IHC. Over the years, the high-throughput technologies allowed the prognosis analysis of the whole transcriptome as well as the integration of non-coding RNA as microRNA and long non-coding RNA showing promising results that still require further validations.

We identified 20% of the 341 reviewed prognostic markers as involved in biological pathways altered in ccRCC of which the main ones are cell cycle, angiogenesis, hypoxia, and immune response. Interestingly, the prognostic immune genes are mainly related to inflammation such as IKBKE that plays a role in regulating inflammatory responses to viral infection (159), the well-known proinflammatory cytokine interleukin-6 (IL6) (160), the complement C5 (161), or the receptor of hyaluronic acid, CD44, involved in inflammation and tissue regeneration (162). These inflammatory markers are mainly related to poor prognosis in agreement with inflammation being a cancer-fueling factor (163).

Among single markers, the most validated markers (KI67, BIRC5, TP53, CXCR4, and CA9) exceed the mere field of ccRCC, except CA9. Two of them, KI67 and BIRC5, were validated on the largest sets of samples, and they are beginning to be used in clinical trials. Among the 13 multiple-marker models proposed in the literature, the most validated ones are the 16-gene assay and the ccrcc1-4 subtyping. Paradoxically, the most famous model, ClearCode34, has been highly validated on several independent datasets, but its clinical utility has not yet been investigated. This review highlights the prognostic molecular predictors that should be investigated in more detail to improve therapeutic care and recommends to focus on the most validated markers or models (KI67, BIRC5, the 16-gene assay, and the ccrcc1-4 subtyping) to be quantified on FFPE samples for an easier clinical use. An important preliminary test should be first to ensure the reproducibility of the quantification on several samples of the same tumor to avoid contradictory conclusions.

Our review has some limitations. Several sources of heterogeneity make difficult the comparison between studies. First, the start points used to compute the survival delays (diagnosis vs. surgery) as well as the type of events (OS, MFS, RFS…) may differ. Another limitation is that we kept markers with a prognostic impact on OS but not RFS or inversely, as well as markers recurrently found to be prognostic even if other studies showed no significance. That is the case of genes mostly studied given their role in the ccRCC carcinogenesis like *VHL* and *BAP1* (164, 165). All the markers proposed in the review were validated in multivariate models in at least two datasets. According to the studies, the multivariate models integrated several genes and/or clinicopathological covariates, but the available clinical annotations strongly differ too. Finally, the increased use of abdominal imaging has resulted in an increase in the number of small renal incidentaloma in recent decades (166). Consequently, the clinical characteristics of the patient cohorts evolved over the 15 years, with an increase of the proportion of early stages. All these heterogeneity sources can explain why some markers can be found to be significantly associated with survival in some studies and not in others. In this context, it is important to note that some markers, notably the ccrcc1-4 molecular subtyping, proved to be related to older therapies, notably antiangiogenic drugs (158), but their predictive power is being considered also for more recent immunotherapies with immune checkpoint inhibitors, as illustrated in clinical trials [BIONIKK trial, NCT02960906 (167)].

In addition to the gene expression markers reviewed here, methylation markers such as the ones that were reviewed by Joosten et al. (168) and mutational markers as the ones reviewed by Mitchell et al. (169) can also be considered and could be integrated in multi-omics prognostic models. Combining the prognostic impact of these omics could improve the accuracy of survival prediction. Altogether, the present comprehensive analysis paves the way to robust and accurate evaluation of the risk of relapse and death for patients with ccRCC.

## Author Contributions

FP: conceptualization, methodology, validation, formal analysis, investigation, resources, data curation, writing—review and editing, and funding acquisition. MA and AR: conceptualization, writing—review and editing, and funding acquisition. WF and CS-F: conceptualization, writing—review and editing, supervision, and project administration. SJ: conceptualization, methodology, validation, formal analysis, investigation, resources, data curation, writing—review and editing, funding acquisition, supervision, and project administration. All authors contributed to the article and approved the submitted version. All authors listed have made substantial, direct, and intellectual contribution to the work and approved it for publication.

## Conflict of Interest

The authors declare that the research was conducted in the absence of any commercial or financial relationships that could be construed as a potential conflict of interest.

## Supplementary Material

Supplementary Table 1 Studies included in the literature review, identified by their PubMed ID. The second column indicates the technologies and the third column the prognostic markers.

Supplementary Table 2 List of the 87 prognostic markers validated on one or more independent studies, identified by the PubMed ID.

Supplementary Table 3 Summary of the 13 multi-marker models.
